# First Dating of a Recombination Event in Mammalian Tick-Borne Flaviviruses

**DOI:** 10.1371/journal.pone.0031981

**Published:** 2012-02-22

**Authors:** Yann Bertrand, Mats Töpel, Annelie Elväng, Wessam Melik, Magnus Johansson

**Affiliations:** 1 Department of Plant and Environmental Sciences, Göteborg University, Göteborg, Sweden; 2 School of Life Sciences, Södertörn University, Huddinge, Sweden; 3 Department of Genetics, Microbiology and Toxicology, Stockholm University, Stockholm, Sweden; Institute of Infectious Disease and Molecular Medicine, South Africa

## Abstract

The mammalian tick-borne flavivirus group (MTBFG) contains viruses associated with important human and animal diseases such as encephalitis and hemorrhagic fever. In contrast to mosquito-borne flaviviruses where recombination events are frequent, the evolutionary dynamic within the MTBFG was believed to be essentially clonal. This assumption was challenged with the recent report of several homologous recombinations within the *Tick-borne encephalitis virus* (TBEV). We performed a thorough analysis of publicly available genomes in this group and found no compelling evidence for the previously identified recombinations. However, our results show for the first time that demonstrable recombination (i.e., with large statistical support and strong phylogenetic evidences) has occurred in the MTBFG, more specifically within the *Louping ill virus* lineage. Putative parents, recombinant strains and breakpoints were further tested for statistical significance using phylogenetic methods. We investigated the time of divergence between the recombinant and parental strains in a Bayesian framework. The recombination was estimated to have occurred during a window of 282 to 76 years before the present. By unravelling the temporal setting of the event, we adduce hypotheses about the ecological conditions that could account for the observed recombination.

## Introduction

The mammalian tick-borne flavivirus group (MTBFG) includes viruses associated with important human and animal diseases such as encephalitis (*Tick-borne encephalitis virus*, TBEV; *Louping ill virus*, LIV; *Langat virus*, LGTV; *Powassan virus*, POWV), hemorrhagic fever (*Omsk hemorrhagic fever virus*, OHFV; *Kyasanur Forest disease virus*, KFDV) and viruses that are not known to be human pathogens (*Royal Farm virus*, RFV; *Karshi virus*, KSIV; *Gadgets Gully virus*, GGYB). They are positive-stranded RNA viruses with a genome of about 10.5 kb that encodes all viral proteins in a single open reading frame (ORF), flanked by untranslated regions (UTRs). The corresponding polyprotein is proteolysed and processed into structural, Capsid (C), Pre-Membrane (PrM), Envelope (E) and nonstructural proteins NS1 (glycoprotein), NS2A, NS2B (protease component), NS3 (protease, helicase, RNA triphosphatase activity and NTPase activity), NS4A, NS4B and NS5 (methyltransferase, RNA-dependant RNA polymerase) [1].

Several viruses bear close evolutionary relationships to TBEV [2]–[6] viz. LIV, Spanish sheep encephalomyelitis virus (SSEV), Turkish sheep encephalitis virus (TSEV) and Greek goat encephalitis virus (GGEV). These four lineages have recently been assigned to a single species dubbed *Tick-borne encephalitis virus* [4], whose members are primarily associated with ixodic hard-tick vectors. Within this species, the TBEV lineage is further divided into three evolutionary distinct subtypes, the Western European- (W-), the Far Eastern- (FE-) and the Siberian- (S-) TBEV [1], [7].

In this contribution, our attention is mainly focused on the evolutionary relationships between W-TBEV, SSEV and LIV. W-TBEV is widely distributed throughout continental Europe and Russia, SSEV is endemic to Spain [2]–[6], [8], and LIV, initially considered to be restricted to the British Isles and Ireland [2]–[6], has now been reported from Norway [2] and Denmark [9], [10]. The ecology and pathogenesis of both W-TBEV and LIV have been intensively investigated [11], [12], whereas studies dedicated to SSEV are scarce.

In contrast to mosquito-borne flaviviruses where recombination events are frequent [13], [14], evolution in the MTBFG was considered to be clonal. This perception changed recently with reports of several putative recombinations in *Tick-borne encephalitis virus* [15], [16]. We aim to investigate the strength of the recombination signals reported by Yun *et al.* [16], since if proved valid their discovery would lead to a radical departure from the classical understanding of the evolutionary dynamic in MTBFG. Although, we could not confirm the previously described recombinations, we did identify a strong recombinant signal in the LIV lineage. Putative parents, recombinant strains and breakpoints were further tested for statistical significance using phylogenetic methods.

The second aspect of this study pertains to date the recombination event. We used the available full length coding genomes for dating, but this small sample may limit the power of the molecular-clock analysis. There are a large number of E-sequences available from molecular epidemiological studies. Unfortunately, E-sequence cannot be used directly to date recombination events, as we estimated that the substitution rates for the E-gene is significantly lower than for other viral genes. This means that dates obtained from E-sequences alone tend to be younger and do not represent accurately the temporal dynamic of this viral lineage. We suggest that a large dataset that includes sonly E-sequences could nevertheless be used to date additional divergence events by specifying informative priors on the ages of some important nodes. We describe an incremental analytical strategy that bases these priors on posterior distributions derived from the analysis of full-length coding sequences following removal of the E-sequences.

## Materials and Methods

### Alignments and sampling

Alignments were generated from GenBank sequences retrieved in January 2011, aligned using Muscle [17], rechecked and improved manually in the UTR regions. Sequences were numbered from the start of the ORFs using Neudoerfl (U27495) as reference. Details on the included sequences are provided in Table S1.

ALN1 contains 41 complete nucleotide sequences of *Tick-borne encephalitis virus* and three out-groups selected among LGTV and OHFV. This initial alignment was scanned for recombination events and then down sampled to an alignment (ALN2) of 28 complete sequences of known collection dates (from 1937 to 2008), with the deletion of out-groups and strains with unusual sampling locations. UTRs and gap columns were deleted. ALN2 was further partitioned by individual genes resulting in alignments ALN2_C, ALN2_PrM, ALN2_E, ALN2_NS1, ALN2_NS2A, ALN2_NS2B, ALN2_NS3, ALN2_NS4A, ALN2_NS4B and ALN2_NS5. Next, we produced ALN3 from ALN2 with the deletion of the E gene and the region of NS3 identified as a possible recombinant fragment. Finally, E_161 was compiled from the 161 longest E-sequences available in Genbank (1033 to 1491 nt in length) endowed with sampling dates (from 1931 to 2008).

### Detection of recombination

An analysis of the entire species (ALN1) was conducted with split networks using the neighbor-net method [18]. Evolutionary distances were estimated using maximum likelihood (ML) with a GTR+Γ_4_+I as the best-fit substitution model as determined by MODELTEST v.3.7 [19], according to the Akaike Information Criterion.

Several methods were used to extract recombination signal from ALN1 with the RDP3beta36 package [20], because inspection of the split network had established the possibility of recombination within the species (see results). All analyses were carried out with Bonferroni correction (*P*-value<0.05) and signals reported by more than one method were retained. RDP [21], GENECONV [22], BootScan [23], MaxChi [24], Chimaera [20], and SiScan [25] were used for screenings the alignment. For this initial phase, the following settings were modified to balance sensitivity and statistical significance: RDP: window size 25, detect recombination between sequences sharing 90% to 100% identity; GENECONV: G-scale 5; BootScan: windows size 100, use NJ trees, 200 bootstrap replicates, cutoff percentage at 95% and Jin and Nei 1990 model; Chimaera: 40 variable sites per window; SisScan: window size 80, slow exhaustive scan. As all methods detected the presence of significant recombinant signals in the NS3 gene, the dataset was further evaluated for phylogenetic evidence of recombination based on an alignment of NS3-sequences derived from ALN1.

### Phylogenetic analyses

For the phylogenetic analysis, the NS3 partitions 5′ and 3′ of the putative recombinant fragment were concatenated. Trees were inferred separately for the recombinant region alone and for the concatenated region.

Maximum likelihood analyses were performed with RAxML VI-HPC v.2.2. [26] via the RAxML Web server [27]. The proportion of invariable sites and the number of bootstrap runs were automatically determined.

Bayesian phylogenetic trees were constructed with a GTR+I+G nucleotide substitution model for the concatenated alignment of NS3 and a GTR+G model for the recombinant partition. Model selection was based on the corrected Akaike information criterion in MrAic [28]. For each alignment, two separate analyses were run simultaneously with MrBayes v.3.2-cvs [29] (source code accessed with CVS 22 January 2009) for 5000000 generations using the default settings for priors and MCMC proposals. Trees were sampled every 1000^th^ generation, and standard deviation of split frequencies was below 0.01 at the end of each analysis. For all Bayesian analyses (i.e. MrBayes and BEAST), mixing of the MCMC chains and effective sample size (ESS) for each parameter estimate were investigated using Tracer v.1.5 [30] which showed convergence and larger than 200 ESS for each summary statistic. For both MrBayes analyses, the first 2500 trees where discarded as burn-in and the 7500 remaining trees were summarized in a majority-rule consensus tree.

For each of the two partitions, we tested alternative topological placement for the putative recombinant strain. Constraining the topology in ML analyses yielded likelihoods for alternative placements that were compared with the likelihood of the best ML tree using the approximately unbiased (AU) test [31] in CONSEL [32]. For this step, ML analyses were performed with PAUP* v.4.0b10 [33] and best trees were sought by heuristic searches (10 random addition replicates, TBR branch swapping, *Multrees* in effect).

Throughout the study, node support was estimated by nonparametric bootstrap (BS, bootstrap support) in ML and with multiple samples from the posterior distribution (PP, posterior probability) in BI.

### Selection analysis

Each separate gene alignment (ALN2_C, ALN2_PrM, ALN2_E, ALN2_NS1, ALN2_NS2A, ALN2_NS2B, ALN2_NS3, ALN2_NS4A, ALN2_NS4B and ALN2_NS5) was investigated for signs of positive selection. To that end, the *dN/dS* ratio for the whole gene, and for each codon in the alignment, was inferred using the M3 model [34] implemented in MrBayes, otherwise using default settings. Mixing of the MCMC chains, as well as the ESS of each estimated parameter was assessed by analyzing the resulting parameter files with Tracer. Each analysis was run until the ESS exceeded 200 for all parameters, after which the probability for the whole gene, or individual codons in the sequence, to have evolved under positive selection was analyzed with Tracer.

### BEAST analyses settings

**Figure 1:**
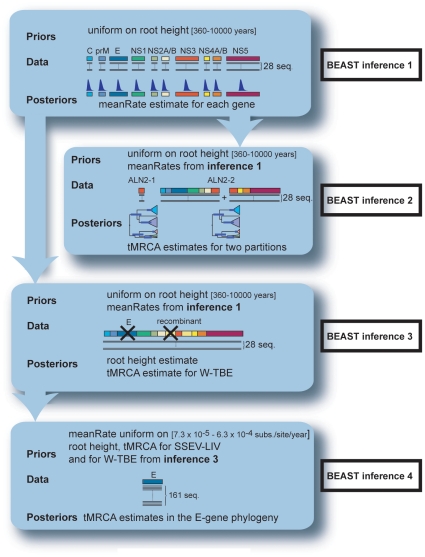
Analytical framework for the BEAST analyses. Inference 1 analyses the variation in substitution rates across the genome for the 28 full length ORFs. The inferred posterior probability distributions of meanRates for individual genes are set as priors for inferences 2 and 3. Inference 2 dates the recombination event based on the 28 full length ORFs. Dates are evaluated separately for the recombinant region and for the non-recombinant sequences. Inference 3 gathers priors information for root height, tMRCA(W-TBE) and tMRCA(LIV & SSEV) used for the next inference. The alignment for inference 3contains the same 28 sequences, with the deletion of E-gene and the recombinant region. Inference 4 refines estimates for tMRCA(W-TBEV) based on 161 E-sequences. Prior distribution for meanRate parameter is derived from the literature. Priors for tMRCA(W-TBEV) and tMRCA(LIV & SSEV) were obtained from BEAST inference 3.

Substitution rates and dates of ancient divergence were estimated with Bayesian MCMC in BEAST version 1.5.3 [35], with collection times in years used as calibration points in the clock model. The youngest strain was collected in 2008, which sets this year as the origin for past time estimates. Each dataset was evaluated individually for best fitting substitution model, which ranged from HKY+Γ_4_+I to GTR+Γ_4_+I. However, analyses performed under GTR family models neither converged nor mixed well, possibly due to an insufficiency of data to estimate these highly parametric substitution models. Hence, the simpler, less parameter rich, HKY+Γ_4_+I model was used throughout the BEAST investigation. We tested the impact of using a GTR model by running an analysis for 20×10^6^ generations. Estimates for the parameters of interest were largely concordant (data not shown), albeit the analyses returned very low ESS and much wider confidence intervals. Pairwise comparisons of Bayes factors calculated in Tracer selected the uncorrelated lognormally distributed relaxed-clock (UCLN) and the Bayesian Skyline coalescence model [36] as the best fitting clock and demographic models following the procedure in Hon *et al.* [37]. We defined two partitions that separated first and second positions from third codon positions. For each analysis, four independent MCMC chains were run for 20×10^6^ generations and their log output combined with 10% burn-in samples discarded. Tracer was used to determine the degree of mixing, shape of the probability density distribution, median and highest posterior density regions at 95% (HPD) for the relevant parameters. The modes and parameters of the posterior distributions were estimated using the distribution fitting software EasyFit 5.3 (MathWave Technology). For all analyzed parameters, we modeled the posterior distributions with gamma distributions. The analytical framework of the BEAST analyses is presented in Figure 1 and the details are explained below.

### BEAST inference 1: Analysis of variation in substitution rates across the genome

We compared the mean substitution rates derived from BEAST analyses for ten individual genes obtained from ALN2. Settings were as described above with an additional uniform prior distribution on the time interval [360–10000] fitted to the root height. This prior captures the background knowledge that crown radiation of flaviviruses occurred after the end of the last glaciations (placed 10,000 years ago) and that the *Tick-borne encephalitis virus* emerged before the divergence of two of its inclusive clades namely LIV and W-TBEV whose split was estimated to be earlier than 360 years ago [38], placing the species divergence within this rather wide interval.

### BEAST inference 2: Dating of the recombination event

To estimate the time of the recombination event (tRE), as well as the time of the most recent common ancestor (tMRCA) for each parental strain, we studied separately the genomic partition spanning the recombinant element from nt 5787 to 5991 (ALN2-1) and the partition covering the rest of the ORF (ALN2-2) that is the 5′ region (nt 1 to 5786) together with the 3′ region (nt 5992 to 10245) flanking the recombinant portion. The same uniform prior was fitted on the root height as before. For individual genes in ALN2-2, prior distributions for the MeanRate parameter were derived from posteriors in BEAST inference 1 with substitution and clock models unlinked during the analysis.

### BEAST inference 3: Gathering prior information for the root height, tMRCA(W-TBE) and tMRCA(LIV & SSEV)

This step was designed to provide posterior distributions for the BEAST inference 4. ALN3 (28 full length ORFs with both E-gene and the recombinant fragment omitted) was analyzed the same way as ALN2-2. The mode and parameters of posterior distributions for the root height, tMRCA(Neudoerfl-Hypr) and tMRCA(LIV & SSEV) were estimated in order to be incorporated as priors in the following step.

### BEAST inference 4: refining estimates for tMRCA(Neudoerfl-Hypr)

Due to its sampling, the E_161 alignment allows access to the antiquity of additional divergence events. Posteriors obtained from BEAST inference 3 were included as priors, with an additional uniform prior distribution over [7.28×10^−5^–6.29×10^−4^ substitutions/site/year] set on the meanRate parameter. This value reflects previously observed substitution rates for the E gene in the *Tick-borne encephalitis virus:* the lower bound comes from the value of 7.28×10^−5^ substitutions/site/year estimated for nonsynonymous substitutions [38], while the upper bound comes from an estimation of 4.78×10^−4^ substitutions/site/year with a standard error of 1.51×10^−4^ [39] for synonymous substitutions. Because the analysis of selection pressure (see results) inferred that a strong purifying selection acts on the proteins, we expect to see higher rate of synonymous substitutions than of nonsynonymous substitutions. As the mean rate takes both types of substitutions into account, its estimate should be intermediate between their two values.

All alignments, xml-files for the BEAST analyses and all phylogenetic trees have been deposited at Dryad Repository: doi:10.5061/dryad.504636cd.

## Results

### Detection of recombination

**Figure 2:**
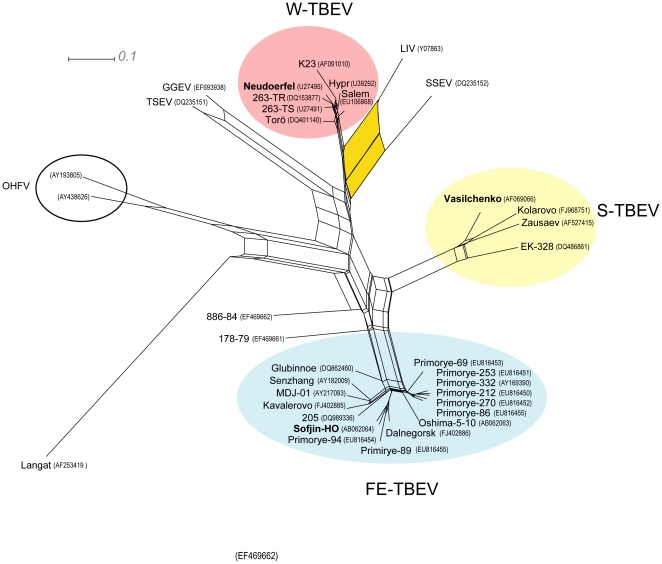
Split-graph constructed by the neighbor-net method based on 41 complete genomes of TBEV, LIV, LGTV and OHFV. The split-graph focuses on phylogenetic relationships within the *Tick-borne encephalitis virus* species. The three TBEV subtypes are highlighted in color and the positions of all prototype strains are indicated in bold. Two Russian strains (886–84 and 178–79) were not assigned to any main subtype at this stage. A larger split between LIV and SSEV (in yellow) suggests a recombination event.

On the inferred network (Figure 2), the region of the split-graph separating the four main clusters exhibits a significant “tree-like” structure that rules out frequent recombination between the clusters. Nevertheless, a prominent split associated with SSEV (DQ235152) and LIV (Y07863) indicates a marked conflicting and/or ambiguous signal that could be associated with a recombination event. This hypothesis was first examined with RDP3, wherein all methods identified the LIV strain as displaying signs of homologous recombination between the SSEV strain as the major parent and a strain belonging to W-TBEV as the minor parent (Figures S1 a–b). All methods recognized with significance that an insert within the NS3 gene of LIV originated from a W-TBEV strain, but they were not consistent with respect to the precise location of the two recombination methods. When run simultaneously, all methods bar, Chimaera and MaxChi, identified Neudoerfl (U27495) as the minor parent and estimated the breaking points at nt 5787 and 5991. When the data were analyzed with Chimaera or MaxChi as single primary detection methods, they instead proposed, with significance (*P*-value <3.10^−2^), slightly different breakpoints (Table 1).

**Table 1:** Localization of the recombination breakpoints with different methods in RDP3 on an alignment of 28 full-length coding genomes, analyzed with Bonferroni correction and *P*-value <0.05.

Detection method	Daughter	Major parent	Minor parent	Av. *P*-Val	Start	End	Size
Genconv	LIV	SSEV	Neudoerfl	1.1×10^−5^	5787	5991	204
Bootscan	LIV	SSEV	Neudoerfl	3.2×10^−6^	5787	5991	204
Chimaera*	LIV	SSEV	Hypr	3.0×10^−2^	5675	6001	326
MaxChi*	LIV	SSEV	Hypr	5.0×10^−3^	5768	6048	280
RDP	LIV	SSEV	Neudoerfl	2.2×10^−3^	5787	5991	204
SiScan	LIV	SSEV	Neudoerfl	8.0×10^−5^	5787	5991	204

Sequences are numbered from the start of the ORF using Neudoerfl as reference.

*indicates that this method did not recover the general recombination signal in a simultaneous run. Instead, it found a different signal when used as a single primary detection method.

No significant evidence for recombination was found in the other strains or genes. We compared our result to the outcome of the screening performed by Yun *et al.* [16] that identified 11 recombinations within the 3′UTR and the 3′ end of the NS5 region, but did not include a LIV strain. Their observations could only be replicated when we used exactly the same settings, i.e. when detection was performed on ClustalW [40] aligned sequences, without Bonferroni correction for multiple comparisons. This suggests that the previously reported signal was not strongly supported and could have been caused by alignment problems, as UTRs are notoriously difficult to align due to spontaneous variations in length during laboratory passages [41], [42].

### Phylogenetic evidence of recombination

**Figure 3:**
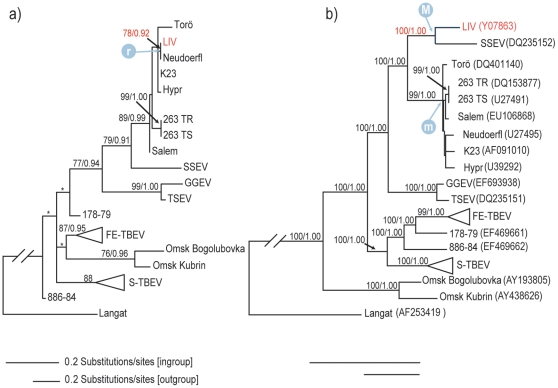
Most likely phylogram from the maximum likelihood analysis of the partitioned NS3-gene. The partitions of NS3 correspond to (a) the recombinant fragment nt 1320–1524, (b) nt 1–1319 concatenated with nt 1525–1866. Numbers above nodes indicate branch support (bootstrap support ≥70%/Bayesian posterior probability ≥0.90). Asterisks (*) mark nodes that that are not recovered by Bayesian inference. Bars show different amounts of substitution for the in-group and out-group taxa. The position and branch support of LIV are indicated in red. “M” and “m” refers to the divergences of the major parent and minor parents respectively, while “r” points to the recombination event in the tree. Genbank accessions are indicated in parentheses.

To evaluate phylogenetic evidence of recombination, trees were constructed for the putative recombinant region and for the concatenated regions of NS3 from both sides of the crossover points. As shown in Figure 3, ML and Bayesian reconstructions contrast the placement of LIV in the two partitions: In the non-recombinant partition, LIV groups with SSEV with maximum support and falls outside the highly supported W-TBEV clade (BS 99%, PP 1.00). In contrast, LIV is well embedded within the W-TBEV clade and is placed together with Neudoerfl for the recombinant partition. Although the two most supported nodes that identify close evolutionary relationships between LIV and a strain from W-TBEV display moderate BS and PP (78% and 0.92 for the branching with Neudoerfl, 89% and 0.99 for the inclusion of LIV within W-TBEV), they are among the most significantly supported nodes in this tree. We tested the three putative recombinant fragments obtained by different methods in RDP3 and found that the shorter segment branched together with Neudoerfl with higher support values. Hence, we proceed with further characterization of this mosaic history under the assumption that crossovers occurred at nucleotides 5787 and 5991, which places the 204 nt long recombination in the highly conserved helicase domain of NS3 (subdomain 3). At the nucleotide level, the comparison of the daughter with its parental strains revealed 23 variable sites within the putative recombinant element, while the rest of the NS3 gene contained 274 variable sites. A comparison of genetic distances based on nucleotide sequence is reported in Table 2.

**Table 2:** Genetic distance between LIV, Neudoerfl and SSEV.

	Recombinant element		Non recombinant part of the genome	
	LIV	Neudoerfl	LIV	Neudoerfl
**Neudoerfl**	0.005, 0.005		0.123, 0.139	
**SSEV**	0.118, 0.158	0.113, 0.151	0.101, 0.111	0.131, 0.148

The first number corresponds to uncorrected distances (p-distance), the second to corrected distances (maximum composite likelihood).

Phylogenetic discrepancies were assessed statistically with the AU test. For the combined (non-recombined) NS3 partition, the topological constraints forced LIV and W-TBEV into a monophyletic group with SSEV as sister taxon. Conversely, for the recombinant partition we imposed the grouping of SSEV and LIV outside the W-TBEV clade. Both alternative placements were rejected by the AU test (see Table 3), confirming that the different placements of LIV between partitions expresses genuine phylogenetic information rather than mere stochastic effects.

**Table 3:** Results of topological constraints and hypotheses testing using the AU test.

Partition	Topological Constraint	*P* AU test
1 (nt 4468–5787)	(SSEV, (LIV, W-TBE))	<0.01
2 (nt 5788–5991)	((SSEV, LIV), W-TBE)	0.025
3 (nt 5992–6333)	(SSEV, (LIV, W-TBE))	<0.01

*P*-value <0.05 indicates rejection or the alternative constrained hypothesis under the AU test.

### Variation in substitution rate across the genome

**Figure 4:**
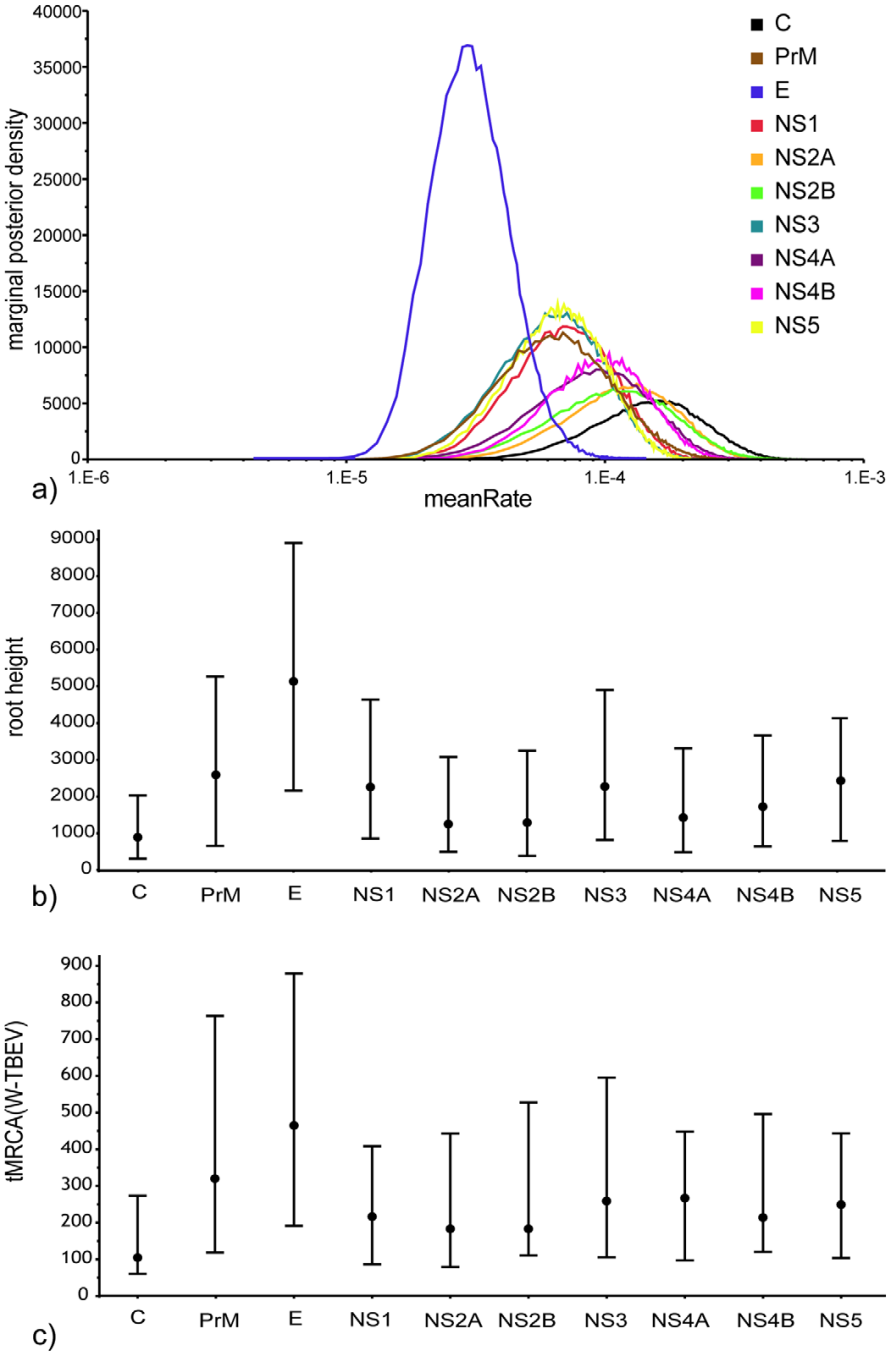
Effect of mean rate variation across the genome and consequences on date estimations. (a) Posterior probability distribution for the mean substitution rate (meanRate) parameter for individual genes across the full-length coding genome. (b) Root antiquity (root height) and (c) tMRCA(W-TBEV) inferred from individual genes for the 28 full-length coding sequences.

Results of BEAST inference 1 are summarized in Figure 4a, showing that under the same set of priors, the posterior substitution rate (meanRate) varies up to five-fold between the different genes. The estimates distinguish the E-gene; it is both the most clearly separated and narrowly distributed, with a median of 3.36×10^−5^ (HPD: 1.5×10^−5^–6.1×10^−5^) substitutions/site/year when compared to the other coding regions, which in contrast span the interval [7.5×10^−5^–3.6×10^−4^]. This analysis discloses a large substitution rate variation across the viral genome. Rate heterogeneity translates into differences in inferred node antiquity (Figure 4b–c). The median estimates for the root age and tMRCA(W-TBEV) range between the inference from the lowest substitutions rate E-gene and those from the highest rate C-gene: The former gene yields 5067 (HPD: 2217–8959) years for the root height and 472 (HPD: 191–879) years for tMRCA(W-TBEV). Conversely, the latter returns the youngest estimates of 939 (HPD: 360–2086) years for the root and 128 (HPD: 60–273) years for W-TBEV divergence.

### Dating the recombination event

In order to increase precision, dating the recombination was carried out using the recombinant region ALN2-1 and the recombination free ORF (ALN2-2). Overall, when compared to the outcome of ALN2-2, divergence times for ALN2-1 were younger and less precise, probably due to the low amount of informative data. For the sake of studying the recombination event, ideally three nodes should be scrutinized: Node “r” on Figure 3 estimates the actual recombination. It refers to the clustering of the LIV recombinant segment with the Neudoerfl strain which places tRE at a median of 76 (HPD: 45–160) years before origin. Accordingly, this time point is paramount and constitutes the lower bound of the estimate, but caution is advised when interpreting it as the definitive estimate. Indeed, it suffers from being inferred from a dataset comprising very short sequences. Moreover this analysis, carried out with a low level of prior enforcement, demonstrates a systematic bias towards younger antiquity. “M” and “m” are time points that refer respectively to the oldest estimate for the emergence of the major and minor parental lineages. Point “M” corresponds to the split of SSEV and LIV lineages, dated at a median of 1017 (HPD: 664 to 1510). Point “m” refers to the emergence of Neudoerfl, which corresponds to its split with the most closely related strain Hypr. However, few substitutions among the nine W-TBEV strains leads to poor phylogenetic resolution. Hence, a more conservative estimate for the onset of the minor parent would coincide with the divergence of the W-TBEV clade, placed at a median of 307 (HPD: 208 to 444) years.

The next step aimed to retrieve divergence times for “M” and “m” with both increased accuracy (better locate the events in time) and increased precision (achieve narrower confidence intervals). We analyzed the largest available dataset for TBEV strains with collections dates (161 sequences); unfortunately, it only covers the Envelope glycoprotein (E) obtained from epidemiological studies. This brings on two problems: Firstly, this dataset is unable to target the actual recombination that occurred within the NS3 gene. Secondly, inference 1 has demonstrated that the E-gene presents the lowest rate of substitution among the viral genes, therefore it estimates older divergence dates than other portions of the genome. Although the former issue cannot be avoided, E-sequences can nevertheless pinpoint which lineages would carry the recombinant element in a much larger tree. The latter issue can be tackled in a Bayesian framework by incorporating posterior information on divergence times derived from full-length coding sequences as prior distributions in an E-sequence analysis. The underlying rational is that by injecting information that pertains to all genes, bar E and the recombinant segment, we would be able to downplay the influence of the low substitution rate, while still combining all available evidence and avoiding circularity. We used BEAST inference 3 to calculate prior distributions for the root age and tMRCA(W-TBEV). The prior on the substitution rate was derived from the literature and not from BEAST inference 1 in order to avoid circularity in the use of data.

**Figure 5:**
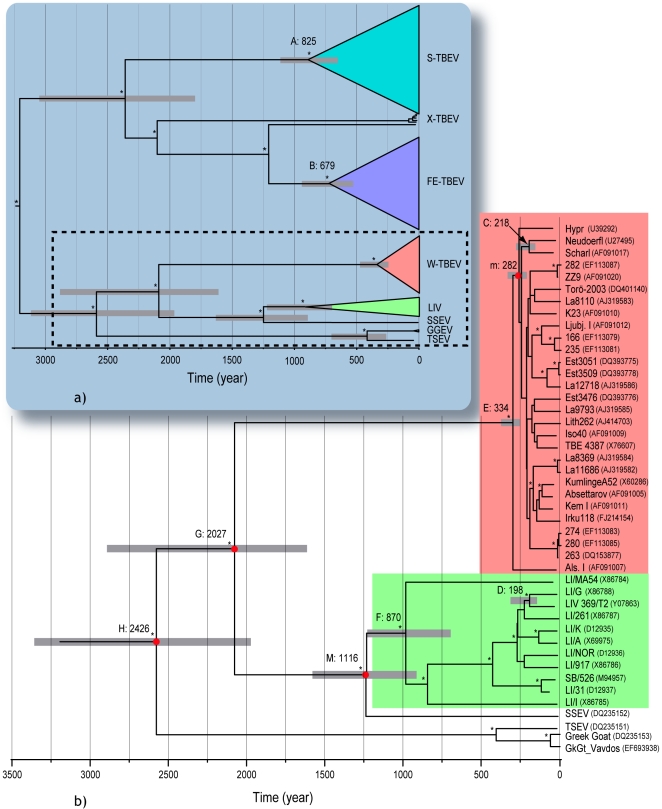
Maximum Clade Credibility tree from BEAST inference 4. The general tree (a) summarizes the entire TBEV species evolutionary history. A paraphyletic group branching at the basis of the far Eastern lineage is addressed by the informal name X-TBEV. It contains Ural Siberian, Central Siberian and Transbaikal strains with characteristic E-gene motifs [61]. The enlarged chronogram (b) focuses on the relationships within W-TBE, LIV, SSEV, GGEV, TSEV cluster. Grey bars at nodes represent 95% HDP credibility intervals. tMRCA are indicated above branches. Nodes investigated for tMRCAs in both analyses of the 163 E-sequences and with the datasets derived from the 28 complete nucleotide genomes are highlighted with red bullets. Asterisks refer to nodes supported by PP>0.90. Nodes with letters are mentioned in the text and in Table 4. Genbank accessions are indicated in parentheses.

Figure 5 depicts the outcome of BEAST inference 4, wherein the general tree summarizes the entire TBEV species evolutionary history and the enlarged chronogram gives median divergence dates within the W-TBEV, LIV, SSEV, GGEV, TSEV cluster. Dates for the principal nodes are indicated in Table 4. Within the cluster concerned with the recombination (Figure 5b), time point C (218 years, HPD: 150–289) refers to the divergence of the two Austrian strains Neudoerfl and Scharl (AF091017), m (282 years, HPD: 228–342) to the divergence of Neudoerfl with Hypr, D (198 years, HPD: 143–263) estimates the divergence of LIV 369/T2 from its closest relativet LI/G (Y07863) and M (1116 year, HPD: 896–1380) the split between LIV and SSEV.

**Table 4:** Times of origin (in years before 2008) for selected clades in the phylogenetic tree of *Tick-borne encephalitis virus*, obtained from the BEAST inference 4 based on a large E-sequences dataset.

Clade	Node in Figure 5b	tMRCA (median)	95% HPD
S-TBEV	A	825	631–1053
FE-TBEV	B	679	505–862
Neudoerfl – Scharl	C	218	150–289
Neudoerfl – Hypr^a^	m	282	228–342
LIV369/T2 - LI/G	D	198	143 to 263
SSEV - LIV^b^	M	1116	896–1380
W-TBEV	E	334	243–442
LIV	F	870	676–1087
W-TBEV - SSEV - LIV	G	2027	1563–2565
W-TBEV - SSEV – LIV – TSEV - GGEV	H	2426	1841–3065

a b correspond to the upper bonds of the divergence times respectively for the minor and major parent identified in figure 3.

tMRCAs estimated from ALN2-2 and E_161 are consistent, suggesting that the appropriate priors have successfully counterbalanced the influence of a lower substitution rate in the E-gene. Inference 2 placed tRE at 76 (HPD: 45–160) years before origin, which localizes the recombination within the Neudoerfl lineage and after the split with the Scharl lineage. The upper (older) bound for the tRE corresponds to the youngest of the parental divergence times in the tree Figure 3. As the estimate for M is much older than time point m, the latter can be considered as the theoretical upper bound for the observed recombination event. The lower bound leaves open the possibility that recombination occurred after the LIV 369/T2 - LI/G divergence, whereas the upper bound sets it within a clade comprising LI/G, LIV 69/T2, LI/261, LI/K, LI/A, LI/NOR and LI/917. Based on previous phylogenetic dispersal reconstructions [39], the first bound places the event in Scotland, whereas the second allows a wide range of locations within the UK, after the initial virus emergence in Ireland.

## Discussion

### Recombination detection

The possibility of recombination within tick-borne flaviviruses was raised by Twiddy *et al.* [14], but given the low amount of genetic variation in this group, they pointed out that detection would prove difficult. A recent report [16] would indicate that tick-borne flaviviruses have the potential to obtain and spread advantageous traits, and to remove deleterious genes [43] by homologous recombination. Alas, re-analysis of the published data did not recover that signal using a more accurate alignment method and more stringent detection conditions, but found evidences for a different event. Therefore our study shows for the first time that demonstrable recombination (that is, with sufficient statistical support and with strong phylogenetic evidences) has occurred in the mammalian tick-borne flavivirus group.

### Mean rate analysis

Substitution rates are compound products of at least four factors: generation time, effective population size, underlying mutation rate and mutation fitness [44]. The last factor can be assessed indirectly by studying the level of selection pressure on the variable sites. The low positive selection is a well documented aspect of the mode of evolution of vector-borne RNA viruses [45]–[47], which demonstrate a lack of immune-driven positive selection [46] and a very effective purifying selection [48]. Our analyses did not identify any site under positive selection. Moreover, the substitution rate analysis yielded a median of 3.3×10^−5^ (HPD: 1.5×10^−5^–6.1×10^−5^) subs./site/year for E, significantly lower than the previously reported rates of 1.6×10^−4^, within S-TBEV [49] and FE-TBEV [50] and the 8.0×10^−4^ found W-TBEV [21]. The main difference with the previous studies can be pinpointed to our use of a relaxed clock, which was chosen because Bayes factor comparisons indicated that the strict clock performed significantly worse than relaxed models. It is known that incorrect clock assumption may lead to spurious rate estimates [51] and dating analyses effectuated under a strict clock and the same set of priors as in inference 1, yielded a mean rate estimate twice as high as under a relaxed clock and, consequently underestimated all divergence times by about a factor two (data not shown).

Woelk *et al.* [47] suggested that the reduced positive selection in vector borne RNA viruses, results from three possible trade-offs associated with the life cycle carried in both mammalian and arthropod hosts: the first posits the presence of non-synonymous mutations that enhance infection or replication in one host, but could have antagonistic effects in the other. The second relates to the differences in replication strategies within the two hosts, with the virus mostly persisting in the tick in a dormant noncytolytic state, while it actively replicates in the mammalian environment. The third addresses the differences in immune response in the two host types: Mutations facilitating immune escape or tolerance in the first host might cause the opposite effect in the second. In the present analysis the Envelope gene displays the lowest substitution rate. As it encodes the protein responsible for the induction of protective antibody response in mammals [52], the reported rate could be explained by the third trade-off mechanism. Accordingly, the other surface-exposed structural proteins do not interact with hosts environment as strongly as the E protein (the M protein is buried under a scaffold of E dimmers and the Capsid is covered by the Envelope) and could accumulate more mutations. We conjecture that the C-gene reaches the highest substitution rates because the Capsid is not directly involved in the replication or in the mounting of an anti-viral immune response. Rate differences for nonstructural proteins could in turn be explained by the first and second trade-offs.

Finally, it has been proposed that rate of replication governs the long-term substitution rate; for instance in dsDNA viruses very high replication rates may inflate the observed substitution rate [53], [54]. Within tick-borne arboviruses, the tempo of replication is the compound of phases of high replication rates following mammalian infection and phases of low to very low rates in the arthropod environment, with the phase transition commanded by a putative termo sensitive ribo-switch [55]. It is unclear how this rate shift would impact our estimate of a global rate and, as opposed to dsDNA viruses, whether the long periods of latency could deflate the observed rates.

### Prior choice and evidence incorporation

The core idea of Bayesian approaches consists in updating our degree of belief in the truth of a hypothesis in light of new pieces of evidence pertaining to it. It is a form of incremental induction wherein the belief at the end of an investigative step is injected as a prior belief for the next step. This new belief will in turn be modified by conditionalizing upon new evidence. In order to reach credibility interval for drawing conclusions about the temporal setting of the RE, we were compelled to apply several informative priors on our final BEAST analysis. In the first step it is beneficial to place a weakly informative prior on the root [56]. This prior obtained from the literature had the effect of concentrating the probability density around its mean so it could be captured by a narrow shaped gamma distribution. In the following steps, formal probability distributions were retrieved from posteriors in the previous step and used as prior assumptions about rates and node antiquity. Although, the overlap of datasets between iterations was kept minimal, our strategy imposes to maintain some sequences across datasets in order to identify the nodes to which the derived prior should be applied. The reduction of the credibility intervals for the date parameters indicates that our approach succeeded to improve the accuracy of the time estimates by combining different lines of information coming from informative data and from the literature.

### Dating

Our use of relaxed-molecular clocks is the main cause for discrepancies between our estimates and previously published divergence dates. Using a strict clock Zanotto *et al.* found four to five times younger divergences than those presently reported (Table 5) [38]. Due to the rejection of the strict clock model, we argue that our approach provides a better estimate of divergence times given the data at hand, although some notes of caution should be raised. Our molecular dating could be hampered by sequencing errors, especially since sequence variation is low. In addition, the low substitution rates, could lead to inaccurate rate estimations [57]. Indeed, our estimates for individual genes approaches the limit of 1×10^−5^ substitutions/site/year below which the temporal signal for heterochronous sampled virus begins to break down [58] and tend not to converge on the true rate when analyzed with BEAST. On that account, the least reliable time estimates are produced by the shortest alignment, which casts doubts on the tRE lower bound that was derived from 204 nt long sequences from the recombinant region. Therefore, although our dating estimates are more accurate than those relying on a poorly fitted molecular clock, more full-length genomes with a wide temporal sampling are required for a definitive assessment of divergence events in the *Tick-borne encephalitis virus*.

**Table 5:** Comparison of divergence times (in years before 2008) for main subtypes in the *Tick-borne encephalitis virus* species between the present study estimates and dating derived from rates of non-synonymous substitution calculated with a strict molecular clock [38].

Divergence event	Present study estimates	Estimates in reference [38]
LI/MA54 & LI/31 (node F)	870 [670–1087]	273 [218–328]
LIV & SSEV (node M)	1116 [896–1380]	268 [214–322]
LIV & W-TBEV (node G)	2027 [1563–2565]	431 [364–498]
LIV & TSEV (node H)	2426 [1841–3065]	449 [379–519]

Node designations refer to Figure 5. Confidence intervals are indicated between brackets.

### Consequences for the evolution of LIV

Our dating locates the tRE after LIV's colonization of the British Isles. Little is known about the modes of *Tick-borne encephalitis virus* dispersal over long distance. Birds on a longitudinal migrating route have been found to carry infected ticks through Scandinavia [59]. However, phylogenetic analyses have not shown any clear admixture of Northern and Southern strains that would point towards bird distribution. Therefore, livestock importation from central Europe to the UK seems a more likely explanation for the footprint of past W-TBEV presence observed in the LIV genome. It is not clear why W-TBEV strains did not form stable foci in the British Isles; possibly the number of continental strains was too small to find its way from infected sheep to the small rodents that are their natural vertebrate hosts.

The ecology of the tick vector, which feeds only occasionally and is relatively immobile, the rarity of infected ticks, implying that the probability of multiple strains co-infecting the same tick must be low, the short mammalian viraemia and high mortality rate, are all plausible factors that would explain that no recombination has hitherto been reported in TBEV [14]. For recombination to occur, a vector can become infected with multiple strains during co-feeding in close proximity on the host skin with other ticks carrying different strains. Co-infection is then mediated via the tick saliva [60]. Alternatively, ticks can engage in multiple feeding on viraemic hosts that have been previously infected with different strains [14]. For both situations, sheep are an ideal milieu for recombination to occur when they are fed upon by several vectors carrying both W-TBEV and LIV strains. Indeed, unlike TBEV, LIV can induce a high-titer viraemia in sheep which enables tick re-infection during bloodsucking [8], [11].

### Conclusion

Given the high similarity between strains within a sub-type, recombinant sequences in *Tick-borne encephalitis virus* species can probably only be detected between sub-types. Dating recombination events is challenging, due to high sequence similarity, low substation rate and condensed temporal sampling. In order to refine this analysis, additional full-length genomes of LIV strains are necessary. Now that the recombining fragment has been identified, it can readily be researched in LIV genomes. Finally, although sequencing the E-gene in order identify strains is a standard practice, the low substitution rate observed in this gene does not supply enough information for robust phylogenetic/phylogeographic studies. We would therefore recommend to sequence, together with E, a faster evolving marker such as the Capsid-gene.

## Supporting Information

Figures S1 **a–b RDP3 analyses results.** The x axis shows genome length in nucleotides, numbered form the start of ORFs after alignment with Neudoerfl (U27495) as reference. The y axis represents the metric used by each method for detecting recombination. Detected recombination signals appear as colored rectangles.
(TIF)

Table S1 **Synoptic table of strains included in the alignments: ALN1, ALN2 and E_161.** Information is provided on strain name, accession, geographical origin, year of isolation, sequence length and assigned clade.
(DOC)
