# Supplementary figures and images for: First Dating of a Recombination Event in Mammalian Tick-Borne Flaviviruses
Source: PLoS One. 2012 Feb 22;7(2):e31981. doi: 10.1371/journal.pone.0031981 (PMC3285191; doi:10.1371/journal.pone.0031981)

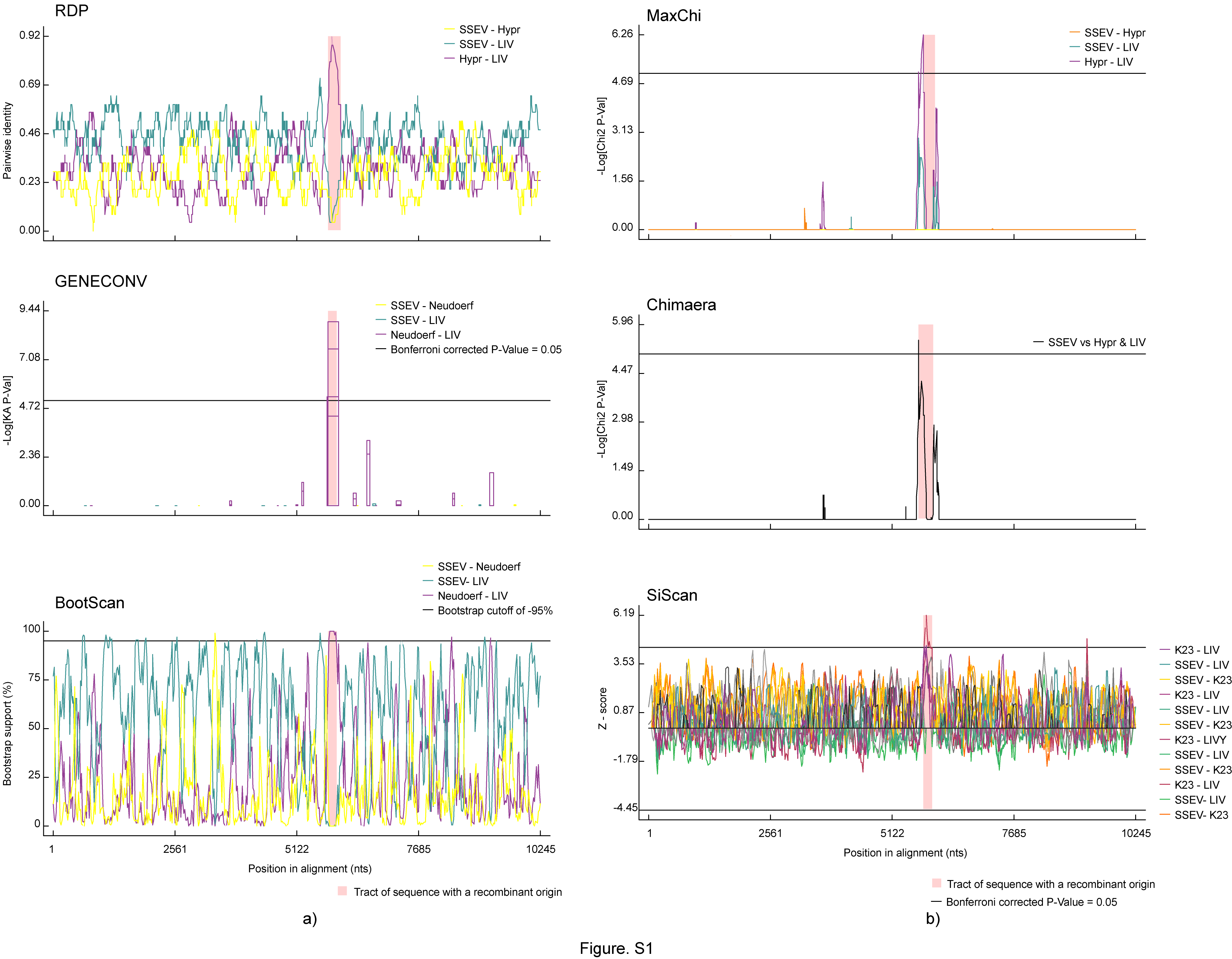

Supplement: Figures S1 — a–b RDP3 analyses results. The x axis shows genome length in nucleotides, numbered form the start of ORFs after alignment with Neudoerfl (U27495) as reference. The y axis represents the metric used by each method for detecting recombination. Detected recombination signals appear as colored rectangles. (TIF) [file pone.0031981.s001.tif]
