# Supplementary material for: First Dating of a Recombination Event in Mammalian Tick-Borne Flaviviruses
Source: PLoS One. 2012 Feb 22;7(2):e31981. doi: 10.1371/journal.pone.0031981 (PMC3285191; doi:10.1371/journal.pone.0031981)
Supplement: Table S1 — Synoptic table of strains included in the alignments: ALN1, ALN2 and E_161. Information is provided on strain name, accession, geographical origin, year of isolation, sequence length and assigned clade. (DOC) [file pone.0031981.s002.doc]

**Table S1.**

**ALN1, 41 strains**

| Strain | Genbank Acc. | Geographical origin | Year of isolation | Length in bp | Clade |
| --- | --- | --- | --- | --- | --- |
| Langat (TP21) | AF253419 | Malaysia | ? | 10943 | LGTV |
| OHFV (Bogoluvovska) | AY193805 | Bogoluvovska, Omsk Russia | ? | 10787 | OHFV |
| OHFV (Kubrin) | AY438626 | Bogoluvovska, Omsk Russia | ? | 10609 | OHFV |
| TSEV | DQ235151 | Turkey | 1980 | 10245 | TSEV |
| GGEV | DQ235153 | Vergina, Greece | 1971 | 10245 | GGEV |
| 263-TR | DQ153877 | Czech Rep. | 2006 | 10923 | W-TBEV |
| 263-TS | U27491 | Czech Rep. | 2006 | 11141 | W-TBEV |
| AS33 | GQ266392 | Amberg, Germany | 2005 | 11097 | W-TBEV |
| Hypr | U39292 | Brno, Czech Rep. | 1953 | 10835 | W-TBEV |
| K23 | AM600965 | Karlsruhe, Germany | 1975 | 10857 | W-TBEV |
| KrM 93 | HM535611 | South Korea | 2006 | 11097 | W-TBEV |
| KrM 213 | HM535610 | South Korea | 2006 | 11097 | W-TBEV |
| Neudoerfl | U27495 | Neudoerfl, Austria | 1971 | 11141 | W-TBEV |
| Salem | EU106868 | Germany | ? | 10871 | W-TBEV |
| Torö | DQ401140 | Torö, Sweden | 2003 | 11094 | W-TBEV |
| LIV (369/T2) | Y07863 | Ayrshire, UK | 1963 | 10871 | LIV |
| SSEV | DQ235152 | Spain | 1987 | 10245 | SSEV |
| EK328 | DQ486861 | Estonia | ? | 10832 | S-TBEV |
| Kolarovo | FJ968751 | Russia | 2008 | 10928 | S-TBEV |
| Vasilchenko | AF069066 | Vasilchenko, Russia | 1969 | 10927 | S-TBEV |
| Zausaev | AF527415 | Russia | 1985 | 10832 | S-TBEV |
| 205 | DQ989336 | Russia | 1973 | 10881 | FE-TBEV |
| Dalnegorsk | FJ402886 | Dalnegorsk, Russia | 1973 | 10881 | FE-TBEV |
| Glubinnoe | DQ862460 | Russia | 2004 | 10886 | FE-TBEV |
| Kavalerovo | FJ402885 | Kavalerovsky, Russia | 1985 | 10875 | FE-TBEV |
| MDJ-01 | AY217093 | China | ? | 10242 | FE-TBEV |
| Oshima5-10 | AB062063 | Oshima, Japan | 1995 | 11100 | FE-TBEV |
| Primorye-18 | GQ228395 | Russia | 1997 | 11096 | FE-TBEV |
| Primorye-69 | EU816453 | Russia | 1969 | 11099 | FE-TBEV |
| Primorye-86 | EU816455 | Russia | 1986 | 10878 | FE-TBEV |
| Primirye-89 | FJ906622 | Russia | 1987 | 10875 | FE-TBEV |
| Primorye-90 | FJ997899 | Russia | 1990 | 10878 | FE-TBEV |
| Primorye-94 | EU816454 | Russia | 1994 | 10984 | FE-TBEV |
| Primorye-212 | EU816450 | Russia | 1991 | 10878 | FE-TBEV |
| Primorye-253 | EU816451 | Russia | 1991 | 10816 | FE-TBEV |
| Primorye-270 | EU816452 | Russia | 1991 | 10878 | FE-TBEV |
| Primorye-332 | AY169390 | Russia | 1991 | 10878 | FE-TBEV |
| Senzhang | AY182009 | China | 1953 | 10245 | FE-TBEV |
| Sofjin-HO | AB062064 | Primorsky krai, Russia | 1937 | 10894 | FE-TBEV |
| 178-79 | EF469661 | Russia | 1985 | 10758 | FE-TBEV |
| 886-84 | EF469662 | Russia | 1979 | 10756 | FE-TBEV |

**ALN2, 28 strains**

| Strain | Genbank Acc. | Geographical origin | Year of isolation | Length in bp | Clade |
| --- | --- | --- | --- | --- | --- |
| TSEV | DQ235151 | Turkey | 1980 | 10245 | TSEV |
| GGEV | DQ235153 | Vergina, Greece | 1971 | 10245 | GGEV |
| 263-TR | DQ153877 | Czech Rep. | 2006 | 10923 | W-TBEV |
| Hypr | U39292 | Brno, Czech Rep. | 1953 | 10835 | W-TBEV |
| K23 | AM600965 | Karlsruhe, Germany | 1975 | 10857 | W-TBEV |
| Neudoerfl | U27495 | Neudoerfl, Austria | 1971 | 11141 | W-TBEV |
| Torö | DQ401140 | Torö, Sweden | 2003 | 11094 | W-TBEV |
| LIV (369/T2) | Y07863 | Ayrshire, UK | 1963 | 10871 | LIV |
| SSEV | DQ235152 | Spain | 1987 | 10245 | SSEV |
| Kolarovo | FJ968751 | Russia | 2008 | 10928 | S-TBEV |
| Vasilchenko | AF069066 | Vasilchenko, Russia | 1969 | 10927 | S-TBEV |
| Zausaev | AF527415 | Russia | 1985 | 10832 | S-TBEV |
| 205 | DQ989336 | Russia | 1973 | 10881 | FE-TBEV |
| Dalnegorsk | FJ402886 | Dalnegorsk, Russia | 1973 | 10881 | FE-TBEV |
| Glubinnoe | DQ862460 | Russia | 2004 | 10886 | FE-TBEV |
| Kavalerovo | FJ402885 | Kavalerovsky, Russia | 1985 | 10875 | FE-TBEV |
| Oshima5-10 | AB062063 | Oshima, Japan | 1995 | 11100 | FE-TBEV |
| Primorye-69 | EU816453 | Russia | 1969 | 11099 | FE-TBEV |
| Primorye-86 | EU816455 | Russia | 1986 | 10878 | FE-TBEV |
| Primirye-89 | FJ906622 | Russia | 1987 | 10875 | FE-TBEV |
| Primorye-94 | EU816454 | Russia | 1994 | 10984 | FE-TBEV |
| Primorye-212 | EU816450 | Russia | 1991 | 10878 | FE-TBEV |
| Primorye-253 | EU816451 | Russia | 1991 | 10816 | FE-TBEV |
| Primorye-270 | EU816452 | Russia | 1991 | 10878 | FE-TBEV |
| Primorye-332 | AY169390 | Russia | 1991 | 10878 | FE-TBEV |
| Sofjin-HO | AB062064 | Primorsky krai, Russia | 1937 | 10894 | FE-TBEV |
| 178-79 | EF469661 | Russia | 1985 | 10758 | FE-TBEV |
| 886-84 | EF469662 | Russia | 1979 | 10756 | FE-TBEV |

**E_161, 161 strains**

| Strain | Genbank Acc. | Geographical origin | Year of isolation | Length in alignment in bp | Clade |
| --- | --- | --- | --- | --- | --- |
| 205 | DQ989336 | Russia | 1973 | 1488 | FE-TBEV |
| Crimea | AF091008 | Crimea, Ukraine | 1987 | 1488 | FE-TBEV |
| DXAL-13 | EU089976 | Heilongjiang Province, China | 2003 | 1488 | FE-TBEV |
| DXAL-16 | EU089978 | Heilongjiang Province, China | 2003 | 1488 | FE-TBEV |
| DXAL-21 | EU089980 | Heilongjiang Province, China | 2003 | 1488 | FE-TBEV |
| DXAL5 | AY178833 | Heilongjiang Province, China | 2003 | 1488 | FE-TBEV |
| Dalnegorsk | FJ402886 | Dalnegorsk, Russia | 1973 | 1488 | FE-TBEV |
| Est2546 | DQ393779 | Estonia | 1996 | 1210 | FE-TBEV |
| Ekaterinburg-Bersenev | FJ214121 | Ekaterinburg, Russia | 1960 | 1419 | FE-TBEV |
| Ekaterinburg-Vinokurov | FJ214120 | Ekaterinburg, Russia | 1969 | 1419 | FE-TBEV |
| Glubinnoe | DQ862460 | Russia | 2004 | 1488 | FE-TBEV |
| Irkutsk-48-06 | FJ214157 | Primorsky krai, Russia | 2006 | 1419 | FE-TBEV |
| Kavalerovo | FJ402885 | Primorsky krai, Russia | 1985 | 1488 | FE-TBEV |
| Kemerovo-Phateev | FJ214132 | Kemerovo, Russia | 1954 | 1419 | FE-TBEV |
| Kemerovo-YuB-40-67 | FJ214133 | Kemerovo, Russia | 1967 | 1419 | FE-TBEV |
| KH98-10 | AB022297 | Khabarovsk, Russia | 1998 | 1488 | FE-TBEV |
| KH98-2 | AB022295 | Khabarovsk, Russia | 1998 | 1488 | FE-TBEV |
| KH98-5 | AB022296 | Khabarovsk, Russia | 1998 | 1488 | FE-TBEV |
| KH99-m9 | AB049346 | Khabarovsk, Russia | 1998 | 1488 | FE-TBEV |
| Khabarovsk-Obor-4 | FJ214111 | Khabarovsk, Russia | 1937 | 1419 | FE-TBEV |
| Kik629/97 | AB237187 | Hokkaido, Japan | 1997 | 1488 | FE-TBEV |
| Kita987/99 | AB237192 | Hokkaido, Japan | 1999 | 1488 | FE-TBEV |
| Kam586/97 | AB237185 | Hokkaido, Japan | 1997 | 1488 | FE-TBEV |
| Kam588/97 | AB237186 | Hokkaido, Japan | 1997 | 1488 | FE-TBEV |
| Miz416/97 | AB237184 | Hokkaido, Japan | 1997 | 1488 | FE-TBEV |
| Miz660/97 | AB237188 | Hokkaido, Japan | 1997 | 1488 | FE-TBEV |
| N132 | AF091013 | Vladivostok, Russia | 1979 | 1488 | FE-TBEV |
| Novo11416 | FJ214158 | Novosibirsk, Russia | 1991 | 1488 | FE-TBEV |
| Oshima 5-10 | AB062063 | Oshima, Japan | 1995 | 1488 | FE-TBEV |
| Oh698/97 | AB237190 | Oshima, Japan | 1997 | 1488 | FE-TBEV |
| Oh701/97 | AB237191 | Oshima, Japan | 1997 | 1488 | FE-TBEV |
| Oh696/97 | AB237189 | Oshima, Japan | 1997 | 1488 | FE-TBEV |
| Oshima I-1 | AB022292 | Oshima, Japan | 1995 | 1488 | FE-TBEV |
| Oshima 3-6 | AB022291 | Oshima, Japan | 1995 | 1488 | FE-TBEV |
| Oshima 5-11 | AB022290 | Oshima, Japan | 1995 | 1488 | FE-TBEV |
| Oshima A-1 | AB022293 | Oshima, Japan | 1995 | 1488 | FE-TBEV |
| Oshima C-1 | AB022294 | Oshima, Japan | 1995 | 1488 | FE-TBEV |
| Primorye-212 | EU816450 | Primorsky krai, Russia | 1991 | 1488 | FE-TBEV |
| Primorye-253 | EU816451 | Primorsky krai, Russia | 1991 | 1488 | FE-TBEV |
| Primorye-270 | EU816452 | Primorsky krai, Russia | 1991 | 1488 | FE-TBEV |
| Primorye-332 | AY169390 | Primorsky krai, Russia | 1991 | 1488 | FE-TBEV |
| Primorye-69 | EU816453 | Primorsky krai, Russia | 1969 | 1488 | FE-TBEV |
| Primorye-86 | EU816455 | Primorsky krai, Russia | 1986 | 1488 | FE-TBEV |
| Primirye-89 | FJ906622 | Russia | 1987 | 1488 | FE-TBEV |
| Primorye-94 | EU816454 | Primorsky krai, Russia | 1994 | 1488 | FE-TBEV |
| RK1424 | AF091016 | Latvia | 1977 | 1488 | FE-TBEV |
| Sofjin-HO | AB062064 | Primorsky krai, Russia | 1937 | 1488 | FE-TBEV |
| T-blood | AF091019 | Perm, Russia | 1939 | 1488 | FE-TBEV |
| Ural-Antipov | FJ214115 | Ekaterinburg , Russia | 1942 | 1419 | FE-TBEV |
| Ural-Belyaeva | FJ214117 | Ekaterinburg , Russia | 1943 | 1419 | FE-TBEV |
| Ural-Ivanova | FJ214116 | Ekaterinburg , Russia | 1942 | 1419 | FE-TBEV |
| Ural-Nina | FJ214119 | Ekaterinburg , Russia | 1943 | 1419 | FE-TBEV |
| Ural-Ponomarev | FJ214118 | Ekaterinburg , Russia | 1942 | 1419 | FE-TBEV |
| Ural-Troynik | FJ214112 | Ekaterinburg , Russia | 1941 | 1404 | FE-TBEV |
| Ural-Yulya | FJ214113 | Ekaterinburg , Russia | 1943 | 1419 | FE-TBEV |
| Volkhov-Khromov | FJ214114 | Volkhov , Russia | 1943 | 1419 | FE-TBEV |
| Yaroslavl-10-89 | FJ214147 | Yaroslavl, Russia | 1989 | 1419 | FE-TBEV |
| VL99-m11 | AB049345 | Vladivostok, Russia | 1999 | 1488 | FE-TBEV |
| Vavdos (Greek Goat) | EF693938 | Vavdos, Greece | 2006 | 1488 | GGEV |
| Greek Goat | DQ235153 | Vergina, Greece | 1969 | 1488 | GGEV |
| LI/261 | X86787 | Newcastle, UK | 1993 | 1488 | LIV |
| LIV (369/T2) | Y07863 | Ayrshire, UK | 1963 | 1488 | LIV |
| LI/MA54 | X86784 | Ireland | 1954 | 1488 | LIV |
| LI/NOR | D12936 | Norway | 1984 | 1488 | LIV |
| LI/31 | D12937 | Thurso, UK | 1931 | 1488 | LIV |
| LI/917 | X86786 | Penrith, UK | 1985 | 1488 | LIV |
| LI/A | X69975 | Devon, UK | 1980 | 1488 | LIV |
| LI/G | X86788 | Mull, UK | 1979 | 1488 | LIV |
| LI/I | X86785 | Aberystwyth, Scotland, UK | 1980 | 1488 | LIV |
| LI/K | D12935 | Grantown-on-Spey, UK | 1980 | 1488 | LIV |
| SB/526 | M94957 | Dublin, Ireland | 1968 | 1488 | LIV |
| 101 | EU443259 | Novosibirsk region, Russia | 1990 | 1172 | S-TBEV |
| 1057 | EU443271 | Novosibirsk region, Russia | 1995 | 1033 | S-TBEV |
| 228 | DQ385498 | Novosibirsk region, Russia | 1981 | 1207 | S-TBEV |
| 323 | EU443263 | Novosibirsk region, Russia | 1988 | 1070 | S-TBEV |
| 668 | EU443260 | Novosibirsk region, Russia | 1992 | 1181 | S-TBEV |
| 902 | EU443261 | Novosibirsk region, Russia | 1994 | 1185 | S-TBEV |
| Aina | AF091006 | Irkutsk region, Russia | 1963 | 1488 | S-TBEV |
| Ekaterinburg-14-5-06 | FJ214125 | Ekaterinburg, Russia | 2006 | 1419 | S-TBEV |
| Est3535 | DQ393774 | Estonia | 2001 | 1210 | S-TBEV |
| Ekaterinburg-35-8-06 | FJ214122 | Ekaterinburg, Russia | 2006 | 1419 | S-TBEV |
| Ekaterinburg-37-3-06 | FJ214126 | Ekaterinburg, Russia | 2006 | 1419 | S-TBEV |
| Ekaterinburg-44-2-06 | FJ214124 | Ekaterinburg, Russia | 2006 | 1419 | S-TBEV |
| Est54 | DQ393773 | Estonia | 2000 | 1210 | S-TBEV |
| Ekaterinburg-56-03 | FJ214127 | Ekaterinburg, Russia | 2006 | 1419 | S-TBEV |
| Irkutsk-112-79 | FJ214156 | Irkutsk . Russia | 1979 | 1419 | S-TBEV |
| IR99-2f13 | AB049353 | Irkutsk, Russia | 1999 | 1488 | S-TBEV |
| IR99-1m1 | AB049349 | Irkutsk, Russia | 1999 | 1488 | S-TBEV |
| IR99-1m4 | AB049349 | Irkutsk, Russia | 1999 | 1488 | S-TBEV |
| IR99-2f7 | AB049352 | Irkutsk, Russia | 1999 | 1488 | S-TBEV |
| Kokkola-102 | DQ451295 | Finland | 2006 | 1225 | S-TBEV |
| Kokkola-118 | DQ451296 | Finland | 2006 | 1225 | S-TBEV |
| Kokkola-26 | DQ451289 | Finland | 2006 | 1225 | S-TBEV |
| Kurgan-264-07 | FJ214130 | Kurgan. Russia | 2007 | 1404 | S-TBEV |
| Kurgan-273-07 | FJ214131 | Kurgan. Russia | 2006 | 1404 | S-TBEV |
| Kurgan-279-07 | FJ214149 | Kurgan. Russia | 2007 | 1419 | S-TBEV |
| Kurgan-316-07 | FJ214151 | Kurgan. Russia | 2007 | 1419 | S-TBEV |
| Kemerovo-K34-67 | FJ214134 | Kemerovo, Russia | 1967 | 1419 | S-TBEV |
| Kurgan-371-07 | FJ214150 | Kurgan. Russia | 2007 | 1419 | S-TBEV |
| Kokkola-39 | DQ451290 | Finland | 2006 | 1225 | S-TBEV |
| Kemerovo-8-11-05 | FJ214135 | Kemerovo, Russia | 2005 | 1281 | S-TBEV |
| Kokkola-84 | DQ451293 | Finland | 2006 | 1225 | S-TBEV |
| Kokkola-8 | DQ451286 | Finland | 2006 | 1225 | S-TBEV |
| Kolarovo-2008 | FJ968751 | Tomsk region, Russia | 2008 | 1488 | S-TBEV |
| Latvia 1-96 | AJ415565 | Latvia | 1996 | 1270 | S-TBEV |
| Vologda-14-06 | FJ214140 | Vologda, Russia | 2006 | 1419 | S-TBEV |
| Vologda-227-07 | FJ214153 | Vologda, Russia | 2007 | 1419 | S-TBEV |
| Vologda-365-75 | FJ214136 | Vologda, Russia | 1975 | 1419 | S-TBEV |
| Vologda-509-75 | FJ214142 | Vologda, Russia | 1975 | 1419 | S-TBEV |
| Vologda-911-74 | FJ214138 | Vologda, Russia | 1974 | 1392 | S-TBEV |
| Vasilchenko | AF069066 | Vasilchenko, Russia | 1969 | 1488 | S-TBEV |
| Volkhov-2-43 | FJ214148 | Volkhov, Russia | 1943 | 1407 | S-TBEV |
| Vologda-3-75 | FJ214143 | Vologda, Russia | 1975 | 1419 | S-TBEV |
| Vologda-4-06 | FJ214139 | Vologda, Russia | 2006 | 1419 | S-TBEV |
| Vologda-658-75 | FJ214137 | Vologda, Russia | 1975 | 1419 | S-TBEV |
| Yaroslavl-115-01 | FJ214145 | Yaroslavl, Russia | 2001 | 1419 | S-TBEV |
| Yaroslavl-140-98 | FJ214146 | Yaroslavl, Russia | 1998 | 1401 | S-TBEV |
| Yaroslavl-2-80 | FJ214144 | Yaroslavl, Russia | 1980 | 1419 | S-TBEV |
| Z 10 | EF566813 | Balahonovka, Russia | 2005 | 1361 | S-TBEV |
| Z 12 | EF566814 | Osinovka, Russia | 2005 | 1361 | S-TBEV |
| Z 14 | EF566815 | Osinovka, Russia | 2005 | 1361 | S-TBEV |
| Z 22 | EF566816 | Tebenka, Russia | 2005 | 1361 | S-TBEV |
| Z 6 | EF566817 | Kemerovo, Russia | 2005 | 1361 | S-TBEV |
| Z 7 | EF566818 | Kemerovo, Russia | 2005 | 1361 | S-TBEV |
| Zausaev | AF527415 | Tomsk, Russia | 1985 | 1488 | S-TBEV |
| SSEV | DQ235152 | Spain | 1987 | 1488 | SSEV |
| TSEV | DQ235151 | Turkey | 1980 | 1488 | TSEV |
| 166 | EF113079 | Czech Rep. | 2006 | 1488 | W-TBEV |
| 235 | EF113081 | Czech Rep. | 2006 | 1488 | W-TBEV |
| 263 | DQ153877 | Czech Rep. | 1987 | 1488 | W-TBEV |
| 274 | EF113083 | Czech Rep. | 2006 | 1488 | W-TBEV |
| 280 | EF113085 | Czech Rep. | 2006 | 1488 | W-TBEV |
| 282 | EF113087 | Czech Rep. | 2006 | 1488 | W-TBEV |
| Absettarov | AF091005 | Russia | 1951 | 1488 | W-TBEV |
| Als. I | AF091007 | France | 1975 | 1488 | W-TBEV |
| Est3051 | DQ393775 | Estonia | 2001 | 1210 | W-TBEV |
| Est3476 | DQ393776 | Estonia | 2001 | 1210 | W-TBEV |
| Est3509 | DQ393778 | Estonia | 2001 | 1210 | W-TBEV |
| Hypr | U39292 | Brno, Czech Rep. | 1953 | 1488 | W-TBEV |
| Irkutsk-118-71 | FJ214154 | Irkutsk, Russia | 1971 | 1419 | W-TBEV |
| Iso40 | AF091009 | Schaffhausen, Switzerland | 1975 | 1488 | W-TBEV |
| K23 | AF091010 | Karlsruhe, Germany | 1975 | 1488 | W-TBEV |
| Kem_I | AF091011 | Tatabanya, Hungary | 1952 | 1488 | W-TBEV |
| KumlingeA52 | X60286 | Kumlinge, Finland | 1959 | 1488 | W-TBEV |
| La11686 | AJ319582 | Latvia | 2001 | 1270 | W-TBEV |
| La12718 | AJ319586 | Latvia | 2001 | 1270 | W-TBEV |
| La8110 | AJ319583 | Latvia | 2001 | 1270 | W-TBEV |
| La8369 | AJ319584 | Latvia | 2001 | 1270 | W-TBEV |
| La9793 | AJ319585 | Latvia | 2001 | 1270 | W-TBEV |
| Lith262 | AJ414703 | Lithuania | 2001 | 1270 | W-TBEV |
| Ljubj. I | AF091012 | Ljubljana, Slovenia | 1993 | 1488 | W-TBEV |
| Neudoerfel | U27495 | Neudoerfl, Austria | 1971 | 1488 | W-TBEV |
| Scharl | AF091017 | Austria | 1956 | 1488 | W-TBEV |
| TBE 4387 | X76607 | Slovakia | 1982 | 1488 | W-TBEV |
| Torö | DQ401140 | Torö, Sweden | 2003 | 1488 | W-TBEV |
| ZZ9 | AF091020 | Zell/Ziller, Austria | 1985 | 1488 | W-TBEV |
| 178-79 | EF469661 | Russia | 1979 | 1488 | X-TBEV |
| 617-90 | EU878283 | Buryat, Russia | 1990 | 1322 | X-TBEV |
| 711-84 | EU878281 | Buryat, Russia | 1984 | 1322 | X-TBEV |
| 740-84 | EU878282 | Buryat, Russia | 1984 | 1322 | X-TBEV |
| 886-84 | EF469662 | Russia | 1984 | 1488 | X-TBEV |
